# Resting-state cerebellar-cerebral networks are differently affected in first-episode, drug-naive schizophrenia patients and unaffected siblings

**DOI:** 10.1038/srep17275

**Published:** 2015-11-26

**Authors:** Wenbin Guo, Feng Liu, Jindong Chen, Renrong Wu, Zhikun Zhang, Miaoyu Yu, Changqing Xiao, Jingping Zhao

**Affiliations:** 1 Mental Health Institute of the Second Xiangya Hospital, Key Laboratory of Psychiatry and Mental Health of Hunan Province, Central South University, Changsha, Hunan 410011, China; 2 Key Laboratory for NeuroInformation of Ministry of Education, School of Life Science and Technology, University of Electronic Science and Technology of China, Chengdu, Sichuan, China; 3 Mental Health Center, the First Affiliated Hospital, Guangxi Medical University, Nanning, Guangxi 530021, China

## Abstract

Dysconnectivity hypothesis posits that schizophrenia is a disorder with dysconnectivity of the cortico-cerebellar-thalamic-cortical circuit (CCTCC). However, it remains unclear to the changes of the cerebral connectivity with the cerebellum in schizophrenia patients and unaffected siblings. Forty-nine patients with first-episode, drug-naive schizophrenia patients, 46 unaffected siblings of schizophrenia patients and 46 healthy controls participated in the study. Seed-based resting-state functional connectivity approach was employed to analyze the data. Compared with the controls, the patients and the siblings share increased default-mode network (DMN) seed – right Crus II connectivity. The patients have decreased right dorsal attention network (DAN) seed – bilateral cerebellum 4,5 connectivity relative to the controls. By contrast, the siblings exhibit increased FC between the right DAN seed and the right cerebellum 6 and right cerebellum 4,5 compared to the controls. No other abnormal connectivities (executive control network and salience network) are observed in the patients/siblings relative to the controls. There are no correlations between abnormal cerebellar-cerebral connectivities and clinical variables. Cerebellar-cerebral connectivity of brain networks within the cerebellum are differently affected in first-episode, drug-naive schizophrenia patients and unaffected siblings. Increased DMN connectivity with the cerebellum may serve as potential endophenotype for schizophrenia.

Schizophrenia is a serious mental disorder with onset commonly during early adulthood and adolescence. In recent decades, schizophrenia is postulated to be a disorder with abnormalities in neuronal connectivity as dysconnectivity hypothesis, which suggests that schizophrenia is a disorder with dysconnectivity of the cortico-cerebellar-thalamic-cortical circuit (CCTCC)^1^,^2^. Dysconnectivity hypothesis posits that abnormal neural connectivity is due to genetic and environmental factors that affect neurodevelopmental process in schizophrenia^3^,^4^. Unaffected siblings have a higher risk to develop schizophrenia than general population, and share similar brain functional abnormalities with the patients^5^,^6^,^7^,^8^,^9^,^10^,^11^, which can serve as potential endophenotypes for schizophrenia. An endophenotype is heritable and segregates with the disorder within families^12^.

Resting-state functional magnetic resonance imaging (fMRI) has observed that intrinsic neural activity across brain regions is organized into functional connectivity (FC) networks^13^. Several high-order networks have been revealed by resting-state fMRI^13^,^14^,^15^,^16^,^17^,^18^. They are: 1) the default-mode network (DMN), a well-known network consisting of the medial prefrontal cortex (MPFC), posterior cingulate cortex (PCC)/precuneus and lateral posterior cortices; 2) dorsal attention network (DAN) including the frontal eye fields, intraparietal sulcus/superior parietal lobule, and extrastriate visual regions; 3) executive control network (ECN) comprising of the dorsolateral prefrontal cortex – parietal regions; and 4) salience network (SN), which includes the anterior cingulate cortex (ACC) and inferior frontal cortex/anterior insular cortex.

Among these networks, the DMN is the most examined network in schizophrenia with mixed findings: increased connectivity^19^,^20^ and decreased connectivity^21^,^22^,^23^ or both^24^,^25^. By contrast, evidence of abnormal connectivity from other networks is limited. For example, several studies have revealed abnormal connectivity within the ECN in schizophrenia^23^,^26^,^27^, whereas Lui *et al.*^28^ reported no changes of the ECN connectivity in first-episode, drug-naive schizophrenia patients. Reduced connectivity in the DMN, DAN and ECN has been found in medicated patients, whereas the SN did not exhibit abnormal connectivity in schizophrenia patients^29^. The mixed findings may result from sample heterogeneity in addition to sample size, scanners and analysis methods. Most of the above-mentioned studies recruited chronic and/or medicated patients. Long illness duration and medication use may have biased their results^30^,^31^,^32^. Therefore, it is important to select first-episode, drug-naive patients as a starting point to reveal the naive connectivity of these networks in schizophrenia.

Furthermore, a critical issue to be settled is whether the connectivity between the above-mentioned networks and the cerebellum is affected in schizophrenia. The cerebellum is traditionally considered as a coordinator of motor function^33^. Evidence of the cerebellum participating high-order brain function has accumulated^34^. Through the CCTCC, the cerebellum connects with widespread cerebral regions^35^. The cerebellum is divided into 26 regions in the Anatomical Automatic Labeling template. Several cerebellar regions have been revealed to link with the cortical networks, i.e., cerebellar Crus I and Crus II with the ECN, lobule VI with the SN, and Crus I, Crus II and lobule IX with the DMN^36^. Reduced cerebellar function and metabolism have been revealed in schizophrenia by neuroimaging studies^33^. Emotional dysregulation and cognitive deficits present in the patients are linked to cerebellar dysfunction, especially with abnormal cerebellar-cerebral connections^37^,^38^. The cerebellum may play a key role in cognition and emotion in schizophrenia patients via the CCTCC^38^. However, it remains unclear to the changes of the cerebral connection with the cerebellum in schizophrenia, especially the cerebellar-cerebral dysconnectivity shared by the patients and the siblings.

In the present study, we explored the cerebellar-cerebral connectivity in a relatively large sample of first-episode, drug-naive schizophrenia patients and unaffected siblings. The FC method was employed to analyze the data using the seeds of the four networks (DMN, DAN, ECN and SN). The aim of this study was to examine the cerebellar-cerebral connectivity shared by the patients and the siblings, which could be used as potential endophenotypes for schizophrenia. According to dysconnectivity hypothesis of schizophrenia, reduced cerebellar-cerebral connectivity was expected to be present in the patients and the siblings. In addition, we examined the correlations between abnormal cerebellar-cerebral connectivity and clinical variables (i.e. symptom severity) in the patients.

## Methods and Materials

### Participants

Forty-nine patients with first-episode, drug-naive schizophrenia patients, 46 unaffected siblings of schizophrenia patients and 46 healthy controls participated in the study. All participants were right-handed, and aged from 16 to 30 years with more than 9 years of formal education. Fourteen patients and 14 unaffected siblings were sib pairs, and the other participants were from different families. The study was conducted in accordance with the Helsinki Declaration^39^. The local ethics committee of the First Affiliated Hospital, Guangxi Medical University approved this study, and all participants gave their written informed consent.

The patients and the siblings were recruited from the Mental Health Center, the First Affiliated Hospital, Guangxi Medical University, China, and the controls were recruited from the community. The siblings had brothers or sisters diagnosed as schizophrenia. The diagnosis of schizophrenia was made based on the Structured Clinical Interview of the Diagnostic and Statistical Manual of Mental Disorders-IV (SCID) criteria, patient edition^40^. The duration of untreated psychosis (DUP) of the patients was less than 3 years, and symptom severity was assessed by Positive and Negative Symptom Scale (PANSS). All participants shared the following exclusion criteria: neurological disorders, severe medical disorders, substance abuse, or any contraindications for MRI scan. The potential controls had a first-degree relative with psychiatric disorders were also excluded.

### Scan acquisition

Scans were acquired on a Siemens 3T scanner. Participants were required to keep still and remain awake with their eyes closed. Soft earplugs and foam pads were applied to reduce scanner noise and head movement. The following parameters with a gradient-echo echo-planner imaging (EPI) sequence were used to obtain resting-state functional scans: repetition time/echo time = 2000 ms/30 ms, 30 slices, 64 × 64 matrix, 90° flip angle, 24 cm field of view, 4 mm slice thickness, 0.4 mm gap, and 250 volumes (500 s).

### Scan preprocessing

Data Processing Assistant for Resting-State fMRI^41^ were employed to preprocess functional scans. After the correction of slice timing and head movement, no participants had more than 2° of maximal rotation and 2 mm of maximal translation. The scans were subsequently normalized to the standard Montreal Neurological Institute (MNI) EPI space in SPM8 and resampled to 3 × 3 × 3 mm^3^. After that, the scans were smoothed with a 4 mm full width at half maximum Gaussian kernel, bandpass filtered (0.01–0.08 Hz), and linearly detrended. Several spurious covariates were regressed, including 24 head motion parameters, signal from a ventricular region of interest (ROI), and signal from a region centered in the white matter. We did not regress out the global signal as previously suggested^42^.

### FC analyses

Seed-based FC method was used to create seed-to-voxel maps for each participant. The seeds were defined as 6 mm radius sphere centered on MNI coordinates applied to detect the corresponding networks in previous studies^18^,^29^,^43^,^44^,^45^. The seeds included: the DMN (1, −55, 17), DAN (left/right: −25, −53, 52/25, −57, 52); ECN (left/right: −42, 34, 20/44, 36, 20), and SN (left/right: −32, 26, −14/38, 22, −10). Software REST^46^ was used to conduct the seed-based FC analyses. Pearson correlation coefficients were computed between the seeds and the voxels of the whole cerebellum to create the correlation maps for each seed and each participant. Finally, the correlation maps were *z*-transformed with Fisher’s *r*-to-*z* transformation.

As described in a previous study^47^, the framewise displacement (FD) was computed for each participant since head micromotion might affect the FC results. The formula for computing the FD was provided in the previous study^47^. For each seed, analyses of covariance (ANCOVA), followed by post hoc *t*-tests, were conducted with the mean FD as a covariate to identify significant differences between each pair of groups. The significance level was set at *p* < 0.005 corrected for multiple comparisons using the Gaussian Random Field (GRF) theory (min *z* > 2.807, cluster significance: *p* < 0.005).

### Correlation analyses

The mean *z* values were extracted from the clusters with abnormal cerebellar-cerebral FC in the patients. Pearson correlations were performed to examine the correlations between the mean *z* values and clinical variables (i.e., symptom severity) in the patients after assessing the normality of the data.

## Results

### Participants

Participant demographics are present in Table 1. The 3 groups do not differ in age, sex ratio, education level, and FD values.

### Seed-based FCs: Group differences

**Figure 1:**
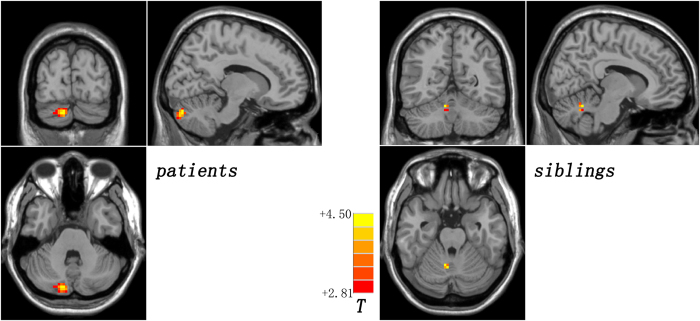
Increased default-mode network seed – right Crus II connectivity shared by the patients and the siblings. Red denotes increased connectivity in the patients/siblings relative to the controls and the color bar indicates *T* values from post hoc *t*-tests.

**Figure 2:**
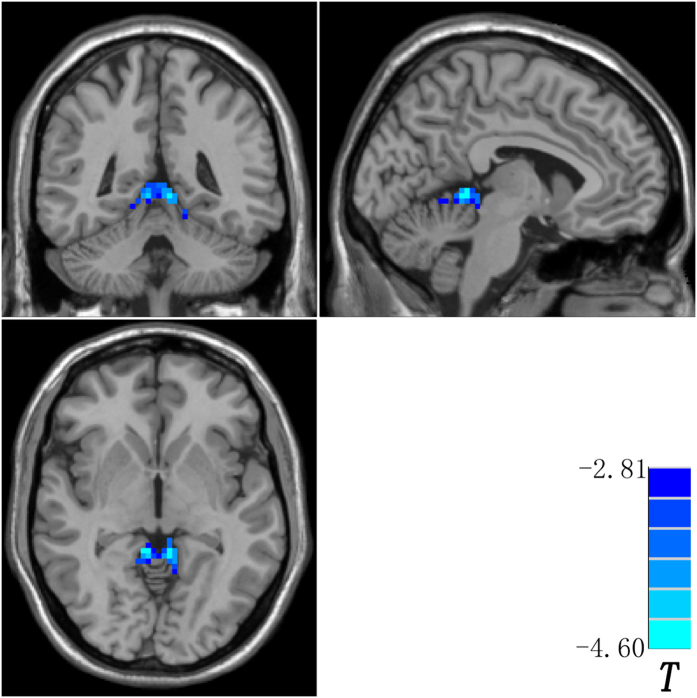
Decreased right dorsal attention network seed – bilateral cerebellum 4,5 connectivity in the patients. Blue denotes decreased connectivity in the patients relative to the controls and the color bar indicates *T* values from post hoc *t*-tests.

**Figure 3:**
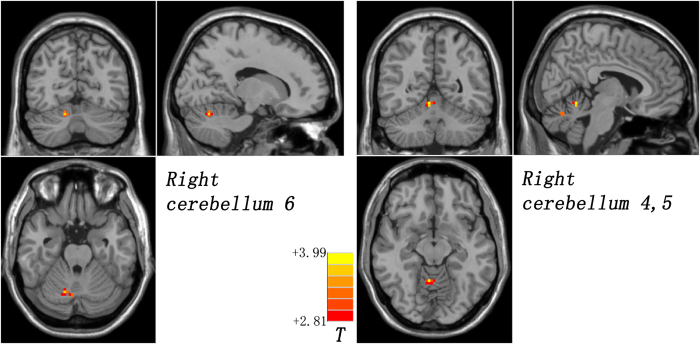
Increased right dorsal attention network seed – right cerebellum 6/right cerebellum 4,5 connectivity in the siblings. Red denotes increased connectivity in the siblings relative to the controls and the color bar indicates *T* values from post hoc *t*-tests.

Compared with the controls, the patients and the siblings share increased DMN seed – right Crus II connectivity by using post hoc *t*-tests (Fig. 1 and Table 2). The patients have decreased right DAN seed – bilateral cerebellum 4,5 connectivity relative to the controls (Fig. 2 and Table 2). By contrast, the siblings exhibit increased FC between the right DAN seed and the right cerebellum 6 and right cerebellum 4,5 compared to the controls (Fig. 3 and Table 2). No other abnormal connectivities (ECN and SN) are observed in the patients/siblings relative to the controls.

### Correlation results in the patients

No correlations are found between the mean *z* values of the clusters with abnormal cerebellar-cerebral connectivities and clinical variables (DUP and PANSS scores) in the patient group. There are also no correlations between the mean *z* values of the clusters with abnormal cerebellar-cerebral connectivities and age or education level in the patients.

### Reproducibility of the shared increased DMN seed – right Crus II connectivity in the patients and the siblings

To examine the reproducibility of the shared increased DMN seed – right Crus II connectivity in the patients and the siblings, the connectivities between the DMN seed, a 6 mm radius sphere centered on the MNI coordinates (1, −55, 17), and other voxels of the whole brain were calculated. Voxel-based one-sample *t*-tests revealed that the DMN seed had links with the cerebellum and the cerebrum in the patients, siblings, and controls (*p* < 0.005, GRF corrected; Figure S1). Then, a DMN mask (Figure S2) was generated from the union of the results of one-sample *t*-tests from 3 groups. Voxel-wise network homogeneity (NH) was calculated with the equation which can be found in a previous study^48^, and the mean NH of each voxel in the DMN mask was obtained and *z*-transformed for standardization purpose. Finally, ANCOVA, followed by post hoc *t*-tests, were computed with the mean FD as a covariate to detect significant NH differences between each pair of groups. Similar results (*p* < 0.005, GRF corrected; Figure S3 and Table S1) were obtained as the original results (Fig. 1 and Table 2).

**Figure 4:**
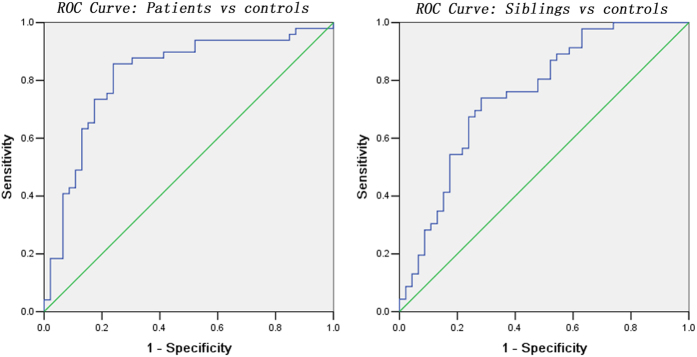
Receiver operating characteristic (ROC) curves using the mean the *z* values of the clusters with shared increased DMN seed – right Crus II connectivity to separate the patients/siblings from the controls. DMN = default mode network.

In addition, mean *z* values of the clusters with shared increased DMN seed – right Crus II connectivity were extracted for further receiver operating characteristic curves (ROC) analysis. As shown in Fig. 4 and Table 3, the areas under the curves were high, which indicated that *z* values of this connectivity might be used as markers to differentiate the patients/siblings from the controls with relatively high sensitivity and specificity.

## Discussion

In the present study, we first examined the abnormalities of the cerebellar-cerebral connectivities in first-episode, drug-naive schizophrenia patients and unaffected siblings using cerebral seeds connecting with the corresponding networks. The main findings were that the patients and the siblings shared increased DMN seed – right Crus II connectivity relative to the controls. Compared with the controls, the patients exhibited decreased right DAN seed – bilateral cerebellum 4,5 connectivity, whereas the siblings had increased right DAN – cerebellum connectivities. No significant correlations were found between the *z* values of abnormal connectivities and clinical variables.

According to the definition, potential endophenotype can be neuroanatomical, neurophysiological, biochemical, endocrine or cognitive parameters. In this study, we observed increased DMN seed – right Crus II connectivity shared by the patients and the siblings. No correlations were observed between the *z* values of this connectivity and clinical variables in the patients, which indicate that this increased connectivity may be a trait alteration for schizophrenia independently of symptom severity and illness duration. Further ROC analysis revealed that *z* values of this connectivity could be used as markers to differentiate the patients/siblings from the controls. From the definition of endophenotype, no correlations to clinical variables and ROC results, increased DMN seed – right Crus II connectivity shared by the patients and the siblings can be used as potential endophenotype for schizophrenia.

At first glance, increased DMN seed – right Crus II connectivity seems inconsistent with the dysconnectivity hypothesis of schizophrenia. The dysconnectivity hypothesis is proposed based on studies with chronic and/or medicated patients^4^. When first-episode, drug-naive schizophrenia patients were recruited in this study, it is not surprised that the present findings were different from those obtained from chronic and/or medicated patients. Increased connectivity can be interpreted from the neurodevelopmental perspective. Previously, the FC differences of the prefrontal-thalamic-cerebellar circuit were examined in healthy children, adolescents, and adults, and the findings showed that the connectivities of the prefrontal-thalamic-cerebellar circuit present an inverted *U*-curve with maximal position in healthy adolescents^49^. Since our patients are at the developmental stage from adolescents to adults (aged from 16 to 30 years), the development process of the DMN seed – right Crus II connectivity is supposed to be disrupted by the disease, and thereby remain at a relatively high position of the inverted *U*-curve. This perspective is supported by previous studies revealing increased frontal FCs in early-course, drug-naive patients^50^,^51^.

Increased connectivity is also meaningful from the physiological perspective of FC. Increased connectivity is usually explained as compensatory reallocation or dedifferentiation^52^,^53^,^54^,^55^. Inflammation may modulate the compensatory reallocation in the early course of the disease^50^. In the early course of schizophrenia, proinflammatory cytokines (i.e., interleukin-6) can activate the astrocytes, and exhibit increased metabolism and blood flow (hyperfunction) to the astrocytes^56^. The regional hypofunction can result in increased regional activity and connectivity. This perspective is supported by a previous study that observed increased connectivities across the DMN areas in early-course patients with schizophrenia^50^. Although we speculated that increased DMN seed – right Crus II connectivity was a compensatory reallocation related to inflammation in the patients, further studies are needed to warrant or to refute this speculation.

The present results are generally consistent with a previous study that reported differentially affected functional resting-state networks in schizophrenia^29^. However, the two studies are different in details. In the previous study, Woodward *et al.*^29^ reported reduced cerebral connectivity in the DMN, DAN and ECN in a group of medicated patients with schizophrenia and schizoaffective disorder. The cerebellum was “cut off” in their study, and we could not compare the two studies directly. In this study, we focused on the cerebral connectivity with the cerebellum, and found increased DMN seed – right Crus II connectivity and decreased right DAN seed – bilateral cerebellum 4,5 connectivity in the patients. In addition to sample size, scanners and analysis method, sample heterogeneity may account for the inconsistency. However, the two studies are consistent when conceived from neurodevelopmental perspective. The patients of Woodward *et al.* had the mean age of 36.9 years and medicated, and the brain connectivity is expected to be at the relatively low position of the inverted *U*-curve. Hence, it is no wonder for their patients to show decreased connectivities.

Interestingly, the siblings exhibit increased DMN and DAN connectivities with the cerebellum. The siblings have a higher risk to develop schizophrenia than the general population although they are unaffected at the recruitment time. The changes of brain connectivity in the siblings suggest that these changes occur earlier than illness onset^57^. Together with the findings from the patients and the siblings and from a previous study^29^, we can portray a picture of the connectivity changes of brain networks for schizophrenia. Increased DMN and DAN connectivities appears earlier than disease onset (as observed in the siblings), and decreases with illness duration. The DAN connectivities decrease immediately after disease onset as reported in our patients.

Previously, associations between abnormal brain function and symptom severity is inconsistent. Positive and negative correlations are reported^23^,^58^, and some studies reported no correlations^59^,^60^. In line with the previous studies^59^,^60^, the present results found no correlations between abnormal cerebellar-cerebral connectivities and clinical variables (DUP and PANSS scores) in the patients. One possibility for no correlations is that abnormal cerebellar-cerebral connectivities are inherent characteristics for the patients independent of the symptom severity and illness duration.

The study has certain limitations. First, we focused on the cerebral connectivities with the cerebellum. This method can enhance the specificity of cerebellar contribution to the neurobiology of schizophrenia. For the same reason, other meaningful information from the cerebrum has been overlooked. Second, only 14 patients and 14 siblings are sib pairs, and the other participants are from different families. This recruitment criterion is critical for the reason that it is different from the traditional sib pair approach. As mentioned in a previous study^61^, recruiting participants from different families can reduce the confounding effect from genes unrelated to schizophrenia and shared early environment. Therefore, the recruitment criterion enhances the specificity of identifying potential endophenotypes for schizophrenia. Finally, this study is cross-sectional. A longitudinal study is needed to precisely portray the connectivity changes of brain networks in the patients and the siblings.

Despite the limitations, this study first observed that the cerebral connectivity of brain networks with the cerebellum are differently affected in first-episode, drug-naive schizophrenia patients and unaffected siblings. Increased DMN connectivity with the cerebellum can serve as potential endophenotype for schizophrenia.

## Additional Information

**How to cite this article**: Guo, W. *et al.* Resting-state cerebellar-cerebral networks are differently affected in first-episode, drug-naive schizophrenia patients and unaffected siblings. *Sci. Rep.* **5**, 17275; doi: 10.1038/srep17275 (2015).

## Supplementary Material

Supplementary Information

## Figures and Tables

**Table 1:** Demographic characteristics of the participants.

	Patients (n = 49)	Siblings (n = 46)	Controls (n = 46)	*p* value
Sex (male/female)	30/19	29/17	23/23	0.39^a^
Age (years)	22.69 ± 4.62	22.96 ± 4.01	23.30 ± 2.30	0.52^b^
Years of education (years)	10.94 ± 2.40	11.50 ± 2.21	11.34 ± 1.78	0.80^b^
FD (mm)	0.06 ± 0.05	0.06 ± 0.03	0.05 ± 0.02	0.15^b^
DUP (months)	22.45 ± 6.71			
PANSS				
Positive scores	22.27 ± 5.33			
Negative scores	22.82 ± 6.86			
Total scores	91.31 ± 10.98			

FD = framewise displacement, DUP = duration of untreated psychosis, PANSS = Positive and Negative Symptom Scale.

^a^The *p* value for sex distribution was obtained by chi-square test.

^b^The *p* values were obtained by analysis of variance (ANOVA).

**Table 2:** Cerebellar regions with abnormal functional connectivity with the cerebral seeds in the patients and the siblings.

Cluster location	Peak (MNI)			Number of voxels	*T* value*
	x	y	z		
*Seed: Default-mode network (1, −55, 17)*					
*Patients *>* Controls*					
Right Crus II	12	−84	−33	47	4.5032
*Siblings *>* Controls*					
Right Crus II	9	−57	−24	11	3.7188
*Seed: Right dorsal attention network (25, −57, 52)*					
*Patients* < *Controls*					
Bilateral Cerebellum 4,5	−6	−45	−3	114	−4.6002
*Siblings* > *Controls*					
Right Cerebellum 6	6	−54	−12	10	3.9930
Right Cerebellum 4,5	15	−66	−24	11	3.7159
*Seed: Left dorsal attention network (*−25, −53, 52)					
None					
*Seed: Executive control network (−42, 34, 20/44, 36, 20)*					
None					
*Seed: Salience network (−32, 26, −14/38, 22, −10)*					
None					

*A positive/negative *T* value represents an increased/decreased functional connectivity; MNI = Montreal Neurological Institute.

**Table 3:** ROC analysis for differentiating the patients/siblings from the controls.

Connectivity	Area Under the Curve	Cut-off point	Sensitivity	Specificity
*Separating patients from controls*				
DMN seed – right Crus II	0.818	0.2209^a^	85.71% (42/49)	76.09% (35/46)
*Separating controls from controls*				
DMN seed – right Crus II	0.751	0.1403	73.91% (34/46)	71.74% (33/46)

ROC = receiver operating characteristic, DMN = default mode network.

^a^By this cut-off point, the *z* values of the clusters with shared increased DMN seed – right Crus II connectivity could correctly classify 42 of 49 patients and 35 of 46 controls, resulted in a sensitivity of 85.71% and a specificity of 76.09%. The meaning of other cut-off point was similar.
