# Supplementary material for: Resting-state cerebellar-cerebral networks are differently affected in first-episode, drug-naive schizophrenia patients and unaffected siblings
Source: Sci Rep. 2015 Nov 26;5:17275. doi: 10.1038/srep17275 (PMC4660304; doi:10.1038/srep17275)
Supplement: Supplementary Information [file srep17275-s1.pdf]

Table S1. Cerebellar regions of the default-mode network with increased network homogeneity in the patients and the siblings

| Cluster location | Peak (MNI) | | | Number of voxels | *T* value |
|---|---|---|---|---|---|
| | x | y | z | | |
| *Patients > Controls* | | | | | |
| Right Crus II | 15 | -84 | -33 | 30 | 4.4486 |
| *Siblings > Controls* | | | | | |
| Right Crus II | 9 | -57 | -24 | 12 | 3.7543 |

MNI = Montreal Neurological Institute

Figure Legends

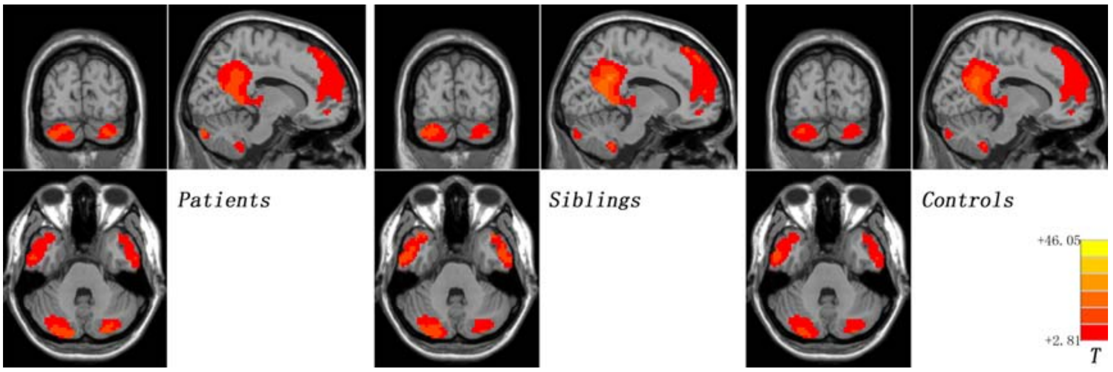


Figure S1. Brain regions having significant connectivity with the default-mode network seed in the patients/siblings/controls. Red denotes increased connectivity and the color bar indicates *T* values from voxel-based one-sample *t*-tests.

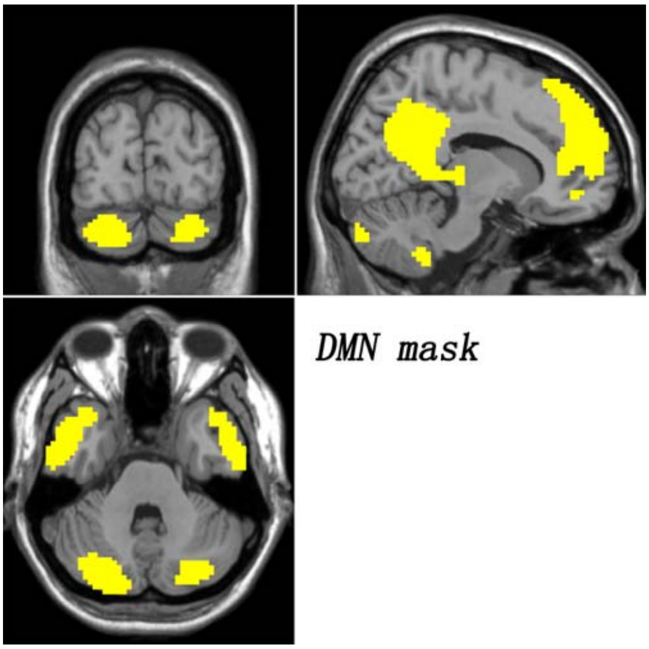


Figure S2. The default-mode network mask generated from the union of the results of one-sample *t*-tests from 3 groups.

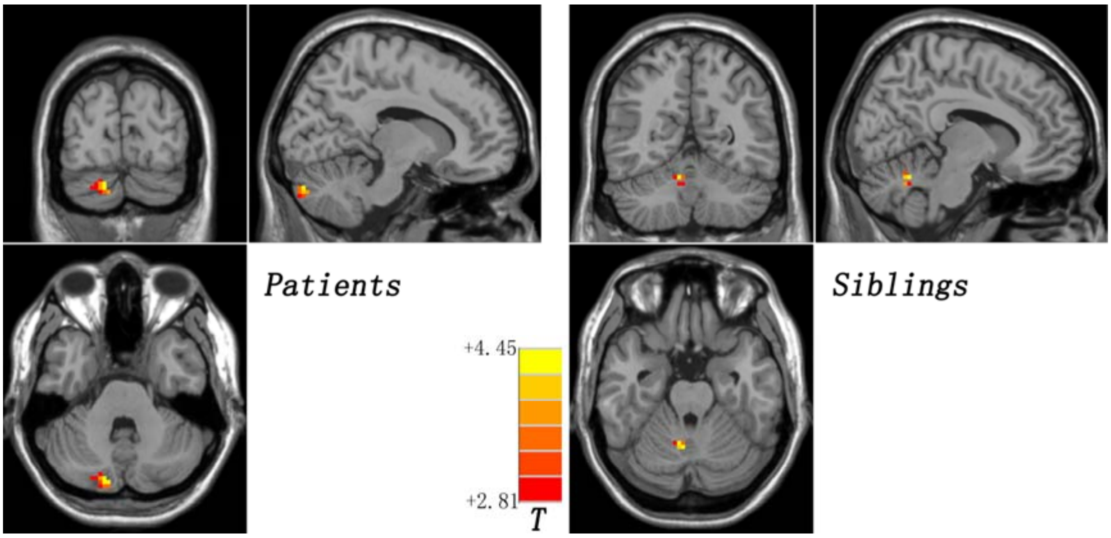


Figure S3. Increased network homogeneity in the right Crus II shared by the patients and the siblings. Red denotes increased connectivity in the patients/siblings relative to the controls and the color bar indicates *T* values from post hoc *t*-tests.
